# Tailoring thixotropic mixed-lipid nanoconstructs of voriconazole for the management of Vulvovaginal candidiasis: Formulation, statistical optimization, *in vitro* characterization and *in vivo* assessment

**DOI:** 10.1080/10717544.2021.1974608

**Published:** 2021-09-14

**Authors:** Wessam H. Abd-Elsalam, Yosra Ibrahim Nagy, Samar M. Abouelatta

**Affiliations:** a Department of Pharmaceutics and Industrial Pharmacy, Faculty of Pharmacy, Cairo University, Cairo, Egypt; b Department of Microbiology and Immunology, Faculty of Pharmacy, Cairo University, Cairo, Egypt; c Department of Pharmaceutics and Industrial Pharmacy, Faculty of Pharmacy, Ahram Canadian University, Cairo, Egypt

**Keywords:** Nanoconstructs, mixed lipids, semisolid, Tefose 63, voriconazole

## Abstract

Vulvovaginal candidiasis is a pervasive gynecological condition among women worldwide due to infection recurrence and resistance to conventional drugs. This calls for a novel formulation of alternative medication and with enhanced efficacy. This study aimed to fabricate mixed-lipid nanoconstructs (MLNCs) of voriconazole (VCZ) with a low concentration of lipids applying high shear homogenization and ultrasonication to form a semisolid formulation. Tefose 63 and Gelot 64 were employed as emulsifiers that are specified for vaginal preparations; as per their mucoadhesive properties and their texture enhancing characters, although usually used as lipids in different lipid carriers. A 2^4^ factorial design was established and the optimized formulation was prepared using 10% total lipids, in which solid lipids (Sterotex NF: Glyceryl monostearate) ratio was 1.92:1 and the oils percentage was 30% (Maisine: Glyceryl monooleate, in the ratio of 1:1), and the emulsifiers mixture (Tefose 63: Gelot 64) ratio was 1:1, as 10% of total formulation weight. The optimized formulation with a viscosity of 964.49 ± 57.99 cp showed spherical nanoparticles (322.72 ± 15.11 nm) that entrapped 67.16 ± 3.45% of VCZ and exhibited release of 70.08 ± 2.87% in 8 h. The optimized formulation with high bioadhesive potentials significantly reduced the fungal burden in female Wistar rats infected with vaginal candidiasis, compared to the aqueous VCZ suspension (*p* < .05). Furthermore, *in vivo* histopathological findings proved the effectiveness and the safety of the optimized MLNCs formulation after vaginal application. Inclusively, MLNCs formulation could be a promising vaginal delivery system of VCZ for the treatment of vulvovaginal candidiasis.

## 1. Introduction

Vulvovaginal candidiasis (VVC) is primarily caused by the yeast Candida albicans (C. Albicans). VVC is considered as a communal fungal infection, where about 75% of women experience at least one episode of the disease during their lifetime and approximately 40–50% will experience a second episode of this infection (Faraji et al., 2012). Different factors contribute to the occurrence of VVC, including; hygienic habits, pregnancy, diabetes mellitus, immunosuppressive diseases, and the administration of hormonal contraceptives, hormone replacement therapy, antibiotics, and steroids (Sardi et al., 2021). The management of VVC involves the use of antifungal drugs, including; polyene antifungal drugs (amphotericin B) and pyrrole ring antifungal drugs (ketoconazole, fluconazole, and itraconazole) (Qin et al., 2018). Fluconazole is the first-line oral antifungal therapy of VVC, but growing rates of resistance and infection recurrency highlight the need for a substitute with enhanced efficacy (Berkow & Lockhart, 2017). Voriconazole (VCZ) is an extended-spectrum triazole antifungal agent and a second-generation synthetic derivative of fluconazole. VCZ is active against a large number of mycoses including Candida, Cryptococcus, as well as other yeasts and hyaline molds. VCZ acts via inhibiting cytochrome P450 dependent enzyme 14-α-sterol demethylase, thus disrupting the sterol biosynthesis pathway and defecting the fungal membrane, and finally inhibits the fungal cell growth and replication. VCZ was also found to inhibit 24-methylene di-hydro-lanasterol demethylation in certain yeast and filamentous fungi, leading to morphologic alterations (Sabo & Abdel-Rahman, 2000; Thompson III & Lewis, 2010). Clinical investigation of the conventional therapeutic modalities of VCZ displayed several side effects; including, nausea, vomiting, and other gastric disturbances (Waghule et al., 2019). As a result, there was a paramount need for the emergence of novel drug delivery systems allowing the reduction of the dose and the alleviation of side effects to improve the clinical efficacy and patient acceptability (Sawant & Khan, 2017). While a formulation for the vaginal delivery of VCZ is unavailable, the efficacy of VCZ by vaginal route in vaginitis using rat model was reported earlier by Gonzalez et al. (2009). In addition, tailoring VCZ nanosystems for vaginal drug delivery is appealing in the avoidance of seepage and messiness, guaranteeing prolonged drug release, and lessening adversative effects, thus promoting patient compliance (El-Hammadi & Arias, 2021).

Nanocarriers are submicron drug vehicles, that represent a breakthrough in the conventional strategies for drug administration. Nanostructured lipid carriers (NLCs) are the following generation of lipid nanocarriers and represent an alternate nanosystem to emulsions, liposomes, and polymeric nanoparticles (Zheng et al., 2013). NLCs or synonymously lipid nanoconstructs (LNCs) (Rehman et al., 2021) are colloidal nanocarriers, self-possessed of spatially incompatible solid and liquid lipids in defined ratios, forming a solid matrix at either room or body temperatures (Mendes et al., 2020). However, most of the research work conducted on LNCs focused on their formulation using single solid lipid and one of the oils; accompanied by the use of conventional surfactants to constitute lipid nanoparticles in aqueous dispersion, a system with an overall low viscosity. Consequently, different approaches emerged to elevate the consistency of LNCs so that they are suitable for topical application. This was accomplished through the addition of the aqueous dispersions to an already prepared gel or cream, or the direct incorporation of viscosity enhancers or gel-forming agents into the aqueous phase, or the production of systems with a high concentration of lipid nanoparticles. Unfortunately, these systems are more complex and suffer from instability problems (Garcês et al., 2018). Nevertheless, a method formulating LNCs with semisolid consistency, containing one lipid and a single oil at 20% total lipid concentration, was previously reported applying homogenization and sonication (Abdel-Salam et al., 2017). To the best of our knowledge, the literature lacks satisfactory data about formulating mixed-lipid nanoconstructs with semisolid texture at low lipids concentrations (10%); that contains a mixture of solid lipids and a blend of oils. Incorporating a mixture of lipids, either solid or liquid, could be beneficial in decreasing the amounts of total lipids used without compromising the viscosity, thus decreasing the greasiness and leakiness; and finally meet the patients’ acceptance and improve compliance. In addition, up to date, no paper reported stabilizing LNCs by emulsifiers, used specifically for vaginal preparations; such as Tefose 63 and Gelot 64, which are usually employed as lipids in the formulation of lipidic nanoparticles.

In the present research work, we investigated the practicability of formulating VCZ loaded mixed-lipid nanoconstructs (MLNCs) with a low lipid concentration and emulsifiers specifically used in vaginal preparations and known for their mucoadhesive properties, customizing high shear homogenization and ultrasonication to form a vaginally applicable dosage form with semisolid consistency, for the treatment of VVC. The influence of different formulation components on the characteristics of MLNCs was studied via a 2^4^ full factorial design by Design-Expert^®^ software. In addition, a histopathological investigation and *in vivo* study were conducted to assess the efficacy and the safety of the optimized formulation in rats infected with vaginal candidiasis.

## 2. Materials and methods

### 2.1. Materials

Voriconazole (VCZ) was provided by Pharco pharmaceuticals (Alexandria, Egypt) as a kind gift. Estradiol valerate was bought from Sigma Aldrich (Germany). Maisine^®^ CC (Glyceryl mono-linoleate), Tefose^®^ 63 (Glycol stearate PEG-6 stearate PEG-32 stearate, HLB = 9.5), and Gelot™ 64 (Glyceryl stearate and PEG-75 stearate, HLB = 10) were obtained from Gattefossé (St-Priest, France). Glyceryl monostearate (GMS), Glyceryl monooleate (GMO), and Sterotex^®^NF (Hydrogenated vegetable oil, type I) were purchased from Abitec Corporation (Columbus, OH). Tween^®^ 80, cellulose membrane tubing (molecular weight cutoff 14,000 Daltons), and mucin were acquired from Sigma-Aldrich, USA. Sodium acetate, glacial acetic acid, and agar were supplied by El-Nasr pharmaceutical chemicals Co. (Cairo, Egypt). Any used chemicals and reagents were of standard grade.

### 2.2. Experimental design and preparation of voriconazole (VCZ) mixed-lipid nanoconstructs (MLNCs)

Before constructing the design, preliminary trials (data are not shown) were conducted employing different solid lipids, surfactants, and oils at different ratios and concentrations. Based on the results of these trials, a 2^4^ full factorial proposal was constructed through Design-Expert^®^ software (Version 7, Stat-Ease Inc., Minneapolis, MN, USA). The proposal investigated the influence of four independent factors at two levels; A: Total lipids percentage in MLNCs at 10 and 20%, B: The solid lipids mixture ratio (Sterotex NF: glyceryl monostearate (GMS)) at 1:1 and 2:1, C: the oils (Glyceryl monooleate (GMO) and Maisine, in ratio 1:1) percentage in lipid phase at 10 and 30%, and D: The emulsifier mixture ratio (Tefose 63: Gelot 64) at 0:1 and 1:1 (10%, w/w of total formulation). The measured responses were; Y_1_: entrapment efficiency percentages (EE %), Y_2_: particle size (PS), Y_3_: viscosity, and Y_4_: the cumulative amount of drug released after 8 h (Q_8_).

MLNCs were formulated as per the method, previously designated by Abdel-Salam et al., utilizing homogenization at high shear and ultrasonication to formulate NLCs semisolid preparation (Abdel-Salam et al., 2017). In detail, the lipid components; namely, GMS and Sterotex NF as solid lipids, and GMO and Maisine as liquid lipids, were allowed to melt together at 60 °C. After that, VCZ (10 mg) was dispersed in the molten lipids. The molten blend was then added portion-wise to the preheated aqueous phase containing the emulsifier mixture (Tefose 63 and Gelot 64) under homogenization with a Silent crusher homogenizer (Heidolph Instrument, Schwabach, Germany) at 26,000 rpm for 5 min. The produced formulations were sonicated for 10 min using a water-bath ultrasonicator (Crest Ultrasonics Corp., NJ, USA). The homogenization and sonication processes were conducted at 60 °C. The VCZ loaded MLNCs formulations were left to cool at room temperature. The compositions of the prepared VCZ loaded MLNCS are listed in Table 1.

**Table 1.** Composition of the prepared VCZ loaded MLNCs (un-coded units) and their observed responses.

#	Factors				Responses*				
	A	B	C	D	Y_1_	Y_2_		Y_3_	Y_4_
	Total lipids (%)	Sterotex NF: GMS	Oils in lipid phase (%)	Tefose 63: Gelot 64	EE (%)	PS (nm)	PDI	Viscosity (cp)	Q_8_ (%)
MLNCs 1	10	1:1	10	0:1	53.25 ± 0.41	241.00 ± 28.85	0.32 ± 0.02	340.50 ± 20.51	97.42 ± 1.54
MLNCs 2	10	1:1	10	1:1	55.98 ± 4.19	226.20 ± 13.01	0.19 ± 0.05	638.50 ± 12.02	99.33 ± 0.45
MLNCs 3	10	1:1	30	0:1	54.68 ± 0.94	325.81 ± 0.01	0.31 ± 0.04	540.00 ± 16.97	94.91 ± 0.47
MLNCs 4	10	1:1	30	1:1	58.59 ± 1.31	235.75 ± 5.02	0.30 ± 0.01	699.50 ± 28.99	97.43 ± 2.34
MLNCs 5	10	2:1	10	0:1	59.20 ± 1.60	357.80 ± 26.30	0.30 ± 0.02	833.00 ± 16.97	77.23 ± 2.60
MLNCs 6	10	2:1	10	1:1	53.28 ± 0.30	345.05 ± 12.80	0.33 ± 0.02	941.00 ± 12.73	71.13 ± 1.38
MLNCs 7	10	2:1	30	0:1	98.74 ± 1.18	407.90 ± 23.48	0.25 ± 0.05	899.50 ± 14.85	65.5 ± 1.27
MLNCs 8	10	2:1	30	1:1	55.00 ± 1.24	374.25 ± 12.37	0.32 ± 0.01	974.00 ± 11.31	64.05 ± 2.73
MLNCs 9	20	1:1	10	0:1	60.84 ± 2.42	333.05 ± 0.07	0.33 ± 0.00	968.50 ± 3.54	71.38 ± 4.32
MLNCs 10	20	1:1	10	1:1	53.09 ± 1.68	249.50 ± 19.52	0.33 ± 0.06	1020.00 ± 7.07	90.67 ± 2.70
MLNCs 11	20	1:1	30	0:1	67.25 ± 1.09	349.75 ± 5.73	0.33 ± 0.01	984.50 ± 3.54	68.92 ± 3.36
MLNCs 12	20	1:1	30	1:1	60.14 ± 1.53	274.40 ± 0.28	0.23 ± 0.03	1199.50 ± 26.16	86.07 ± 3.66
MLNCs 13	20	2:1	10	0:1	42.69 ± 0.82	533.85 ± 9.97	0.31 ± 0.04	1490.00 ± 84.85	60.05 ± 1.10
MLNCs 14	20	2:1	10	1:1	78.61 ± 1.32	340.80 ± 9.48	0.24 ± 0.07	2630.00 ± 0.00	46.05 ± 2.44
MLNCs 15	20	2:1	30	0:1	87.55 ± 1.35	743.95 ± 16.48	0.30 ± 0.07	1877.50 ± 74.25	48.32 ± 4.53
MLNCs 16	20	2:1	30	1:1	84.28 ± 1.59	365.20 ± 6.79	0.21 ± 0.01	2830.00 ± 169.71	40.77 ± 4.55

MLNCs: mixed-lipid nanoconstructs; GMS: Glyceryl monostearate; EE%: Entrapment efficiency percentage; PS: particle size; PDI: polydispersity index; Q8: the cumulative percent of VCZ released after eight hours. VCZ (10 mg) was incorporated in all formulations.

*Values are represented as mean ± standard deviation (*n* = 3).

### 2.3. Characterization of VCZ loaded MLNCs

#### 2.3.1. Entrapment efficiency percentages (EE %) measurements

Owing to the high viscosity of MLNCs, the samples were diluted with water at the ratio of 1:5 and vortexed for 10 min, then shaken within an incubator shaker (Unimax, IKA, Germany) at 200 rpm for 15 min. Afterward, the mixture was subjected to centrifugation at 10,000 rpm and 4 °C (Sigma 3–30 KS, Sigma Laborzentrifugen GmbH, Germany) for 45 min. The supernatant was separated and passed through a 0.2 mm Millipore-filter membrane, then the amount of VCZ was determined spectrophotometrically at a wavelength of 256 nm (UV-1601 PC Shimadzu spectrophotometer, Kyoto, Japan). The measurements were carried out in triplicate. The entrapment efficiency was calculated as follows:

(1) $$\text{EE}\;\%=\frac{\text{Amount of VCZ initially added}-\text{Amount of VCZ determined}}{\text{Amount of VCZ initially added}}\times 100$$

#### 2.3.2. Particle size (PS) and polydispersity index (PDI) analysis

Zetasizer Nano ZS (Malvern Instruments, UK) was used to determine the mean particle size (PS) and the polydispersity index (PDI) of VCZ loaded MLNCs formulations. Prior to the measurements, samples were adequately diluted with bi-distilled water and vortexed for 30 s. All determinations were conducted in triplicate.

#### 2.3.3. Viscosity measurement

The rheological measurements of the formulations were determined using Anton Paar^®^ viscometer (Anton Paar^®^ GmbH, Ostfildern, Germany). The procedure was conducted on a 0.5 g sample of the formulations at 25 ± 2 °C, where the rpm was increased from 0.5 to 350 with 20 s between every two consecutive speeds, while the shear rate ranged between 10 and 450 sec^−1^. The shear stress and the viscosity values were recorded at different shear rates not including torque values less than 10% and more than 100%. All measurements were conducted in triplicate.

#### 2.3.4. *In vitro* drug release study

The release study of VCZ from MLNCs formulations and the aqueous suspension was conducted in an incubator shaker (Unimax, IKA, Germany). Based on the entrapment efficiency percentages, a sample of each MLNCs containing the equivalent to 2 mg of VCZ was packed into a cellulose membrane tubing; tightly closed at both ends. Before conducting the study, the cellulose membrane tubings were drenched in bi-distilled water overnight. On the study day, the loaded cellulose tubings were immersed into the release medium; 50 mL acetate buffer (pH 4, simulating vaginal pH) containing 0.5% Tween^®^ 80 (to maintain sink conditions) at 37 ± 0.5 °C for 8 h at 50 rpm. Samples (2 mL) were withdrawn at specified time intervals and an equal volume of the fresh medium was added immediately to retain the volume unchanged. The aliquots were analyzed spectrophotometrically at λ_max_ 256 nm (UV-1601 PC Shimadzu spectrophotometer, Kyoto, Japan) and the cumulative amounts of drug released versus time plots were constructed. All determinations were conducted in triplicate.

### 2.4. Optimization of the design

The optimized MLNCs formulation was chosen by applying the desirability function. The optimized MLNCs formulation was selected on basis of minimizing total lipids % and maximizing Tefose 63: Gelot 64 ratio. The dependant variables; PS, viscosity, and Q_8_ were minimized, while EE % was maximized. The optimized MLNCs formulation was prepared according to the predicted levels of formulation factors and characterized to confirm the validity of the optimization procedure, Table 2.

**Table 2.** The composition and the observed and the predicted values of the optimized VCZ loaded MLNCs formulation.

Factor	Optimized level (*D* = 0.863)
A: Total lipid (%)	10 %
B: Sterotex NF: GMS	1.92:1
C: Oils in lipid phase (%)	30 %
D: Tefose 63: Gelot 64	1:1

Response	Expected	Observed^#^	Residual*
Y_1_: EE%	65.79	67.16 ± 3.45	–1.37
Y_2_: PS (nm)	328.61	322.72 ± 15.11	5.89
Y_3_: Viscosity (cp)	952.54	964.49 ± 57.99	–11.59
Y_4_: Q_8_ (%)	66.66	70.08 ± 2.87	–3.42

D: Desirability value; GMS: Glyceryl monostearate; EE%: Entrapment efficiency percentage; PS: particle size; Q8: the cumulative percent of VCZ released after eight hours.

*Residual = expected value – observed value.

#Observed values are presented as mean ± standard deviation (*n* = 3).

### 2.5. Characterization of the optimized formulation

#### 2.5.1. Determination of EE %, PS, and PDI

The measurement of EE% was performed as mentioned earlier under Section 2.3.1., while PS and PDI determinations were carried out according to the procedure formerly described under Section 2.3.2.

#### 2.5.2. Transmission electron microscopy (TEM)

TEM was used to envision the microstructure of the optimized VCZ loaded MLNCs. A pre-weighed amount of the formulation was added to 5 mL bi-distilled water and vortexed, then 50 µL of the dispersion was loaded on a metallic grid. The grid was left in the air at room temperature to dry, afterward, it was inspected and simultaneously photographed by the transmission electron microscope (Joel JEM 1230, Tokyo, Japan).

#### 2.5.3. Ph measurements

The pH value of the optimized VCZ loaded MLNCs formulation was determined via a digital pH meter (Jenway, UK) at 25 °C. A pre-weighed amount of the formulation was added to 5 mL bi-distilled water and vortexed, the electrode was immersed into the dispersion until a fixed measurement was recorded. The procedure was performed in triplicate.

#### 2.5.4. Rheological and spreadability testing

The viscosity measurement was performed as per the procedure mentioned earlier in Section 2.3.3. In addition, the flow behavior of the optimized formulation was considered as per Farrow’s equation:

(2) Log D=N Log S−Log g

where D is the shear rate (sec^−1^), S is the shear stress (Pa), N is the flow index (Farrow’s constant), and g is the viscosity (cp).

The value of (N), if equals to one, then the flow follows Newtonian behavior, while values less than one designate shear rate thickening flow, and values more than one, are characteristic to shear rate thinning systems (Abouelatta et al., 2018).

The spreadability was determined according to the method reported earlier by Gollavilli et al., (2020). The optimized VCZ loaded MLNCs formulation (1 g) was placed between parallel glass slides, then a weight of 200 g was situated on the upper slide for 60 sec to uniform the thickness. Accordingly, the maximum area to which the formulation was able to spread was determined via the equation:

(3) S=(W.A)/T

where, S = spreadability (g.cm.sec^−1^), W = weight (200 g), A = the spreading area of the formulation and T = time (60 sec).

#### 2.5.5. Bioadhesion test

The bioadhesion of the optimized VCZ loaded MLNCs formulation was verified as per the technique reported by Nakamura et al. (1996). In brief, 50 g of preheated (60 °C) agar-mucin in phosphate buffer (pH = 6) was transferred to a plate, at a concentration of 1%–2% w/w. The mixture was left to solidify for 3 h at 4–6 °C. The plate was then left to equilibrate at 25 °C and 75% relative humidity in a specified chamber. One gram of the optimized VCZ loaded MLNCs formulation was situated on the surface of the plate, which was then hanged in a vertical position for 24 h. The distance (cm) moved by the formulation was determined. The distance measured proportionates reciprocally to the bioadhesivity of the formulation. The procedure was performed in triplicate.

#### 2.5.6. *In vitro* drug release study

The *in vitro* drug release study was conducted as previously mentioned under Section 2.3.4. The release profile of the optimized VCZ loaded MLNCs formulation was plotted and the data were fitted to four different kinetic models, namely; zero-order, first-order, Higuchi, and Korsmeyer Peppas using the Excel add-in software package DDSolver as follows:

(4) $$\text{Zero}-\text{order}:\;Q=Q_{0}+K_{0}t$$

(5) $$\text{First}-\text{order}:Q=\;\text{Log}\;\;Q_{0}-K_{1}t/2.303$$

(6) $$\text{Higuchi}:Q_{t}=Q_{H}t^{0.5}$$

(7) $$\text{Korsmeyer Peppas}:\;\text{Log}\;\;Q_{t}/Q_{\infty }=\text{n Log t}+\text{Log k}$$

#### 2.5.7. Stability study

The physical stability of the optimized VCZ loaded MLNCs formulation was evaluated after being stored for 3 months in a tightly capped container at refrigerator (6 ± 2 °C), and room temperature (25 ± 2 °C). The instability of the formulation was assessed by the visual observation and the reinspection of EE %, PS, PDI, rheological and spreadability measurements, pH, and Q_8_. Statistical analysis was performed using paired student t-test and statistical significance was considered at *p* < .05.

### 2.6. *In vivo* study

#### 2.6.1. Animals

The protocol of the study was performed according to the established ethical and regulatory guidelines of the Research Ethics Committee, Faculty of Pharmacy, Cairo University, Egypt. (PI 2607). The study was conducted on twenty-five female Wistar rats; weighing 80–100 g. The animals were kept in cages and fed with a standard diet (ad libitum) with permitted access to water. The rats were housed in an air-conditioned chamber (25 ± 0.5 °C) under a controlled relative humidity of 65%. Before the study, the rats were medically checked to ensure their normal physical state and lack of any clinical abnormalities.

#### 2.6.2. Inoculum preparation

*C. albicans* ATCC 60193 was used to induce vaginal candidiasis in rats. The inoculum size was adjusted to an optical density (OD_600_ = 1) corresponding to a cell count of 1 × 10^6^ CFU/mL. The cell count was verified by adopting a 10-fold serial dilution viable count experiment.

#### 2.6.3. Induction of vaginal candidiasis

Vaginal candidiasis was induced according to the method described by Salah et al. (2018) with some modifications. Briefly, the rats were injected subcutaneously with 100 µL estradiol valerate (4 mg/mL) to induce a pseudo-estrus cycle, 48 h before the inoculation. In addition, each animal was intraperitoneally injected with 0.15 mg dexamethasone, 24 h before inoculation, and 24 and 48 h after inoculation. The infection was induced by the intravaginal inoculation of the animals with 250 µL saline suspension containing 1 × 10^6^ CFU/mL *Candida albicans* for three consecutive days. The rats were kept in a supine position for 5 minutes after each inoculation procedure. At the end of the third day, vaginal swabs were taken to confirm the occurrence of candidiasis.

#### 2.6.4. Experimental design

Five uninfected rats were placed in group I (negative control), while the infected rats were equally and randomly allocated into four groups (*n* = 5). Group II was left untreated (positive control), group III received the optimized drug-free MLNCs formulation, group IV was treated with VCZ aqueous suspension, and group V: was treated with the optimized VCZ loaded MLNCs formulation. On the fourth day of the experiment, 0.25 g sample, with VCZ dose of 2.5 mg/Kg of body weight (Gonzalez et al., 2009; Deshkar & Palve, 2019), was applied into the vaginal cavity with a soft plastic tip of a micropipette and a vaginal swab was taken at time intervals 24, 48 and 72 h after the vaginal application of the treatments. The cotton end of each swab was cut and suspended in sterile saline (1 mL), which was then vortexed. A volume of 10 µL of serially diluted microbial suspension (10-fold) was mottled on the surface of Sabouraud dextrose and incubated for 48 hours at 37 °C. The colony-forming units (CFU) of candida were counted and the records were stated as log_10_ CFU/mL. At the end of the experiment, the animals were euthanized, decapitated and vaginal samples were collected for the histopathological examination.

#### 2.6.5. Histopathological examination

Vaginal samples were flushed with saline and then fixed in 10% formol saline for 72 hrs. The tissues were cut, treated with serial dilutions of alcohol, cleared in xylene then entrenched into Paraplast wax. A rotatory microtome was used to cut sections of tissue (4 µm), which were then placed on glass slides. Hematoxylin and Eosin were used to stain the tissues. All data and micrographs were obtained by a full HD microscopic camera operated by the Leica application module (Leica Microsystems GmbH, Wetzlar, Germany). The procedures for sample treatment and staining were carried out as per the methods described by Culling (Culling, 2013). The tissues were examined thoroughly and lesion scoring was performed for all groups.

#### 2.6.6. Statistical analysis of data

The results are stated as average values ± standard deviation. Two-way analysis of variance (ANOVA) was applied to test the statistical significance at *p* < .05, then the Bonferroni post hoc test was applied via SPSS software 17.0 (SPSS Inc., Chicago, USA).

## 3. Results and discussion

### 3.1. Fabrication of VCZ loaded MLNCs

MLNCs were prepared using 10–20% total lipids, applying high shear homogenization and ultrasonication. A mixture of solid lipids (Sterotex NF: GMS), at 90% and 70% w/w of the total lipids, was incorporated in the formulation, while 10 and 30% w/w of the lipids was replaced with the oil mixture (GMO and Maisine, in the ratio of 1:1). The lipids were emulsified by a mixture of Tefose 63 and Gelot 64 at a total concentration of 10% w/w of the formulation. VCZ was added at a constant concentration of 0.1% w/w of the total formulation.

### 3.2. Analysis of the factorial design

#### 3.2.1. Effect of independent variables on EE %

**Figure 1.**
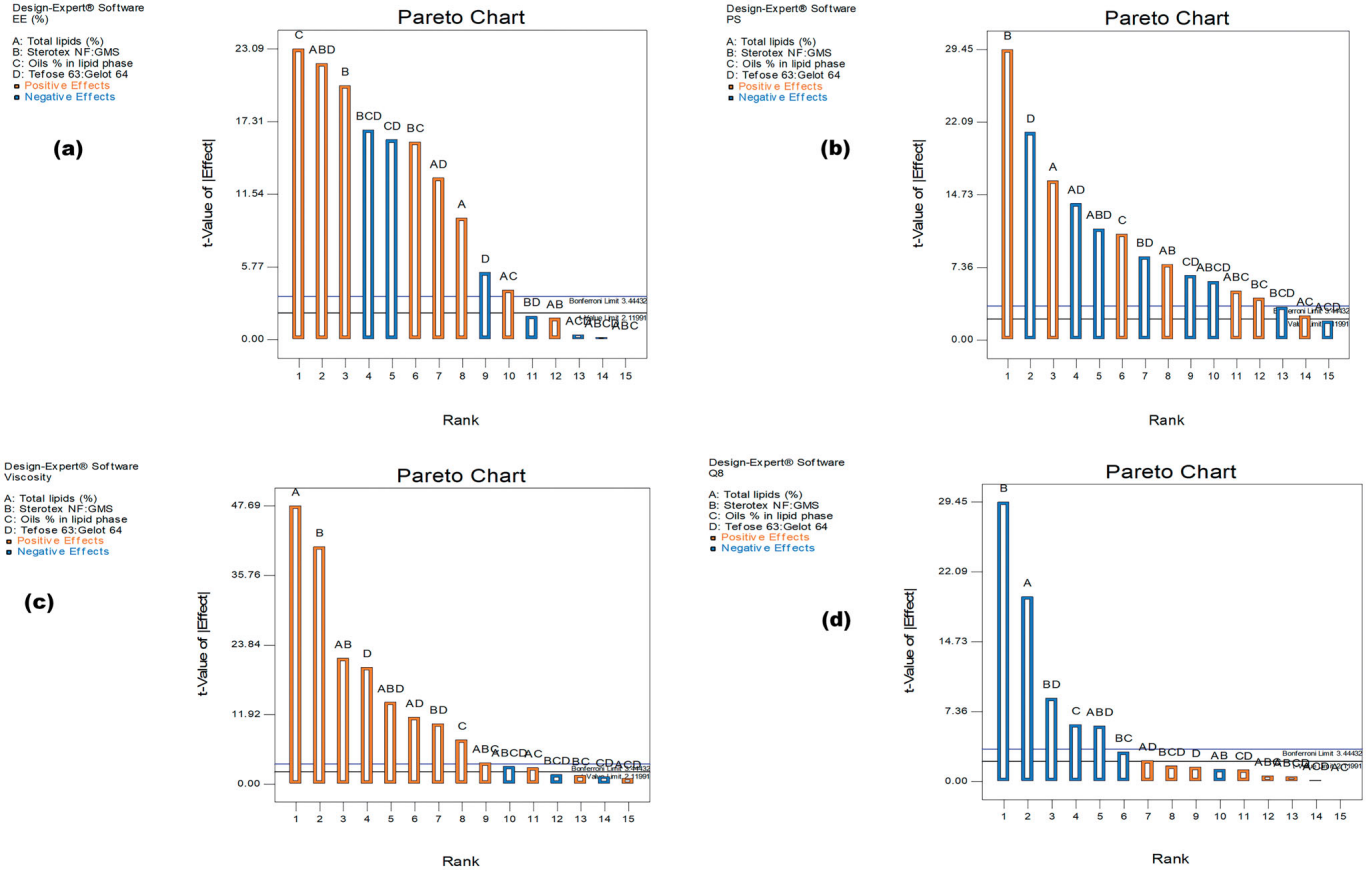
Pareto charts demonstrating the effect of the factors; A: Total lipids percentage, B: The solid lipids mixture ratio (Sterotex NF: glyceryl monostearate (GMS)), C: the oils (Glyceryl monooleate (GMO) and maisine, in ratio 1:1) percentage in lipid phase, and D: The emulsifier mixture ratio (Tefose 63: Gelot 64) on (a) entrapment efficiency percentages (EE %), (b) particle size (PS), (c) viscosity and (d) the cumulative amount of drug released after 8 h (Q_8_).

The percent of VCZ entrapped in MLNCs is considered a crucial parameter for their application as a vaginal dosage form. The EE % was found to be in the range of 42.69 ± 0.82 to 98.74 ± 1.18% as shown in Table 1. The Pareto charts, shown in Figure 1(a), revealed that the total lipids (%) (A), the solid lipids mixture ratio (Sterotex NF: GMS) (B), and the oils percentage in lipid phase (C), had a significant synergistic effect on EE % (*p* < .05), whereas the emulsifier mixture ratio (Tefose 63: Gelot 64) (D) had an antagonistic effect (*p* < .05). The total lipids percentage plays a profound role in lipid-based nanoparticles, where higher percentages result in the formation of a larger core matrix, thus creating more space for drug accommodation, and so a higher amount of drug become entrapped (Firdaus et al., 2021). These outcomes were coinciding with that of Van-An Duong et al. during the formulation of ondansetron hydrochloride NLCs (Duong et al., 2019). Moreover, increasing the lipid percentage reduces the time required for preparation, which in turn minimizes the leakage of the drug to the aqueous phase, and therefore maximizes the percent of drug entrapped (Duong et al., 2020). Regarding the solid lipid mixture ratio, it was observed that decreasing the amount of GMS (at a higher ratio of Sterotex NF: GMS) in the preparation increased the percent of VCZ entrapped. This can be discussed in light of GMS surfactant-like character, where it could have increased the solubility of the hydrophobic drug and permited its escape from the core matrix (HLB of GMS =3.8) (Liu et al., 2016). The positive effect observed upon increasing the percent of oils on the amount of drug entrapped was quite predictable and goes with previous findings of Abuosamra & Mohsen (2016). The authors deduced that the presence of liquid lipids with solid lipids reduced the crystallinity, and thus increased the crystal lattice imperfections, letting more amounts of the drug get entrapped, and subsequently enhancing drug entrapment efficiency. The emulsifier mixture ratio (Tefose 63: Gelot 64) showed an inverse effect on EE %, where the introduction of Tefose 63, aided Gelot 64 in improving the solubility of the hydrophobic drug in the external phase resulting in lower entrapment percentages. A mixture of emulsifiers with comparable HLB values possesses a higher emulsification power than each one of them solely. In addition, Tefose 63 is known to form more stable systems in comparison to Gelot 64, which means the formation of smaller PS and hence lower EE % (Deshmukh & Amin, 2013).

#### 3.2.2. Effect of independent variables on PS

In most VVC cases, recurrent infection was clinically observed as the hyphae of fungi pierce the mucus lining of a vagina. The nanosized particles perform an important role in mucus permeation, retention, and bioadhesion so that they were found promising in minimizing the recurrence of the infection (Firdaus et al., 2021). PS of MLNCs ranged from 226.20 ± 13.01 to 743.95 ± 16.48 nm as shown in Table 1. Upon statistical analysis, Pareto charts in Figure 1(b) showed that all the studied factors have a significant effect on PS (*p* < .05). The total lipids (%) (A), the solid lipids mixture ratio (Sterotex NF: GMS) (B), and the oils percentage in the lipid phase (C) were found to have positive effects on PS, while the emulsifier mixture ratio (Tefose 63: Gelot 64 ratio) (D) was found to have a negative influence on PS. Increasing the total lipids percentage in MLNCs from 10% to 20% resulted in a significant increase in PS. This could be a reflection of the developed viscosity imparted by the higher amounts of added lipids, leading to reduced homogenization efficacy and increased particle agglomeration (Sanad et al., 2010, Abuosamra & Mohsen, 2016). Besides, elevated drug EE % observed with the high level of total lipids %, could be a factor in increasing PS, where more amounts of the drug were internalized inside the particles, resulting in the formation of larger particles (Yu et al., 2016). Using a higher ratio of Sterotex NF: GMS resulted in the formation of MLNCs with larger PS. This could be rationalized in terms of the chemical structure and molecular mass, where Sterotex NF has a complex molecular structure with a relative molecular mass of 1500, while GMS is a simpler molecule with a molecular mass of 358.57 g/mol, and in this case, the higher molecular weight and amount of Sterotex NF produced MLNCs with larger PS. Moreover, as mentioned earlier, GMS has certain surface activity, and decreasing its amount resulted in higher PS (Liu et al., 2016). The oils employed; namely, Maisine and GMO, increased PS significantly, and this could be discussed in light of their chemical structure, where both are considered as esters of long-chain fatty acids (C_16_–C_22_) (Sek et al., 2002). Long-chain glycerides could increase the viscosity of the lipid mixture, unlike medium-chain glycerides, which are known to lower the viscosity of the lipid mixtures and thus decrease PS (Jakubiak et al., 2017). On the other hand, a higher emulsifier mixture ratio (Tefose 63: Gelot 64 ratio) (D) resulted in a smaller PS. This might be explained by the heightened steric stabilization achieved by the dense hydrophobic tail of both emulsifiers when compared to Gelot 64 solely, thus preventing aggregation of nanoparticles and yielding smaller PS (Rehman et al., 2021). Moreover, Tefose 63 played a role in decreasing EE % through lowering PS, as mentioned earlier under Section 3.2.1 (Deshmukh & Amin, 2013).

The values of PDI ranged from 0.19 ± 0.05 to 0.33 ± 0.01 demonstrating a fine distribution of particle size as depicted in Table 1. This is quite attributed to the effectiveness of the preparation method that employed homogenization and ultrasonication (Duong et al., 2020). Besides, the appropriate selection of emulsifiers type and amount that yields relatively small PS with narrow size distribution.

#### 3.2.3. Effect of independent variables on viscosity

The viscosity of vaginal MLNCs is a paramount factor that determines the spreadability of the formulation and hence its contact with the infectious microorganisms (*C. Albicans*) causing VVC, and the diffusion of VCZ from the lipid matrix which in turn would facilitate its flux (Kenechukwu et al., 2018). The viscosity of the prepared formulation (at 100 rpm) was observed to be from 340.50 ± 20.51 to 2830 ± 169.71, Table 1. The Pareto chart in Figure 1(c) stated that all the studied factors were synergistically affecting the viscosity (*p* < .05). Increasing the concentration of total lipids from 10% to 20% significantly increased the viscosity of MLNCs, through the production of larger particles. These results were previously noted by Gonzalez-Mira et al. and Irina Pereira et al., where too viscous preparations were achieved when the total lipid content exceeds 10% forming semisolid preparations (Gonzalez-Mira et al., 2011; Pereira et al., 2018). Herein, the increase in Sterotex NF resulted in the formulation of MLNCs with higher viscosities. This could be discussed in light of the increased PS of the formulated MLNCs due to the high molecular weight of Sterotex NF. Increasing the percent of oils from 10% to 30% increased the viscosity. This might be attributed to the enlarged PS of the formulation because of the long-chain tails of Maisine and GMO, which might have increased the thickness of the whole preparation. Finally, the emulsifier mixture ratio (Tefose 63: Gelot 64 ratio) (D) was found to increase the viscosity of the prepared formulations. Using surfactants of high HLB values leads to the formation of a soft preparation that accomplishes the creation of highly viscous mixed crystal bilayer networks with a high number of emulsified particles in a compact closely packed three-dimensional network. Also, there is a possibility of the formation of a separate crystalline lipophilic network in formulations comprising high concentrations of emulsifier, which could lead to an increase in the viscosity of the system and therefore a higher degree of physicochemical stability of the cream formulation (Fauzee & Walker, 2020). It is worthy to mention that, the addition of Tefose 63 played a dual role as an emulsifier and as a mucoadhesive agent. Being a mucoadhesive agent, Tefose 63 increased the viscosity, and thus the retention of the formulation is supposed to increase; therefore, minimizing the leakage and prolonging the contact time, and enhancing the treatment efficacy (Bolla et al., 2020).

#### 3.2.4. Effect of independent variables on Q_8_

**Figure 2.**
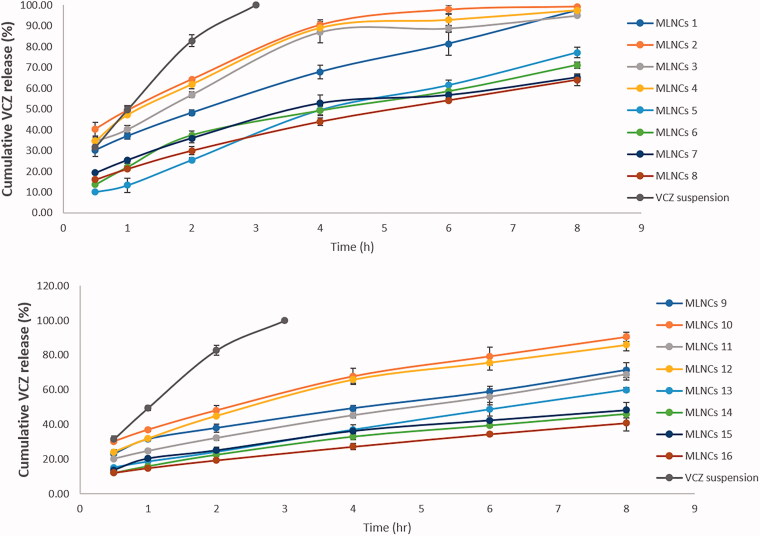
Release profiles of VCZ from the prepared MLNCs and VCZ aqueous suspension in acetate buffer (pH 4, simulating vaginal pH) containing 0.5% Tween^®^ 80 at 37 ± 0.5 °C.

The knowledge of the release profile of the drug is particularly important as the produced information gives a prediction about the *in vivo* behavior of the dosage form. The cumulative percent of VCZ released from MLNCs after 8 hours ranged from 40.77 ± 4.55 to 99.33 ± 0.45%, Figure 2. Upon analyzing the factorial design, the Pareto charts, shown in Figure 1(d), revealed that all the studied factors negatively affected the percent of drug released after 8 hours (*p* < .05) except for the emulsifier mixture ratio (Tefose 63: Gelot 64) (D) which was nonsignificant (*p* > .05). Increasing the percent of total lipids (A) from 10% to 20% led to a significant decrease in the amount of drug released due to the embedment and entrapment of the drug in the solid lipid matrix, where the diffusion of the drug was retarded (Youssef et al., 2020). Similar findings were formerly reported by Niculae et al. (2013). Incorporating an increased ratio of Sterotex NF: GMS sustained the drug release, and this might be due to the increased consistency of the formulation, due to the high molecular weight of Streotex NF compared to GMS. Increasing the percentage of oils retarded VCZ release from MLNCs, which might be accredited to the higher viscosity and increased thickness of lipid layer, which acted as a barrier in the way of the drug, thereof sustaining its release (Gujjar et al., 2019). The results suggested that VCZ is incorporated in MLNCs and this guarantee its slow release to prolong the contact time and so maximizing the efficacy (Wang et al., 2017).

### 3.3. Optimization of the design

**Figure 3.**
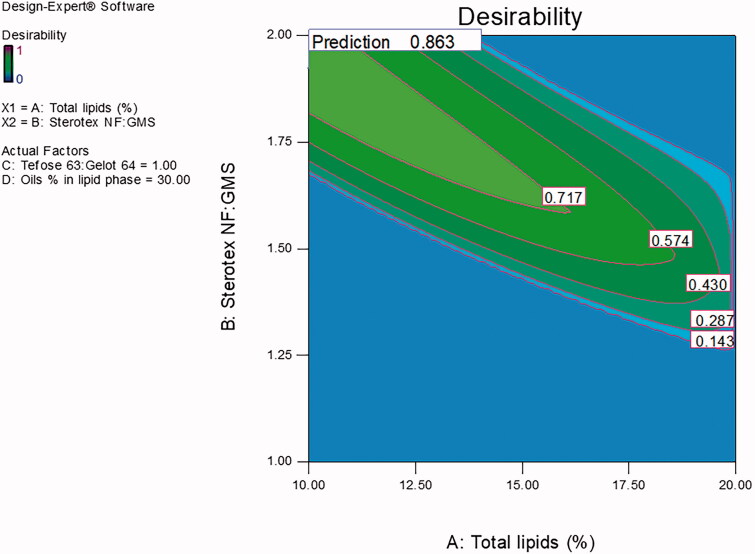
The desirability value graph of the optimized VCZ loaded MLNCs formulation.

Desirability function, using Design Expert^®^ software, was employed to achieve the optimized formulation with minimal total lipids % and maximal Tefose 63: Gelot 64 ratio. The criteria set for the optimized formulation were minimum PS and viscosity with maximum EE % and releasing about two-thirds of its drug content in 8 h. With a desirability value of 0.863 (Figure 3), the optimized formulation was prepared using 10% total lipids, the ratio of Sterotex NF: GMS was 1.92:1, the oils % of the lipid phase was 30% (Maisine: GMO, in the ratio of 1:1) and the emulsifiers mixture (Tefose 63: Gelot 64) ratio was 1:1 at 10%, w/w of the total formulation. The optimized formulation was found to be a new formulation; not one of the design points, therefore a full characterization of the new formulation was performed.

### 3.4. Characterization of the optimized MLNCs

#### 3.4.1. Ee %, PS, and PDI

The optimized VCZ loaded MLNCs showed an EE % of 67.16 ± 3.45%, while the measured PS was 322.72 ± 15.11 nm, Table 2. The formulation exhibited a PDI of 0.22 ± 0.03. In addition, the validity of the design was checked by calculating the residuals as shown in Table 2.

#### 3.4.2. Tem

**Figure 4.**
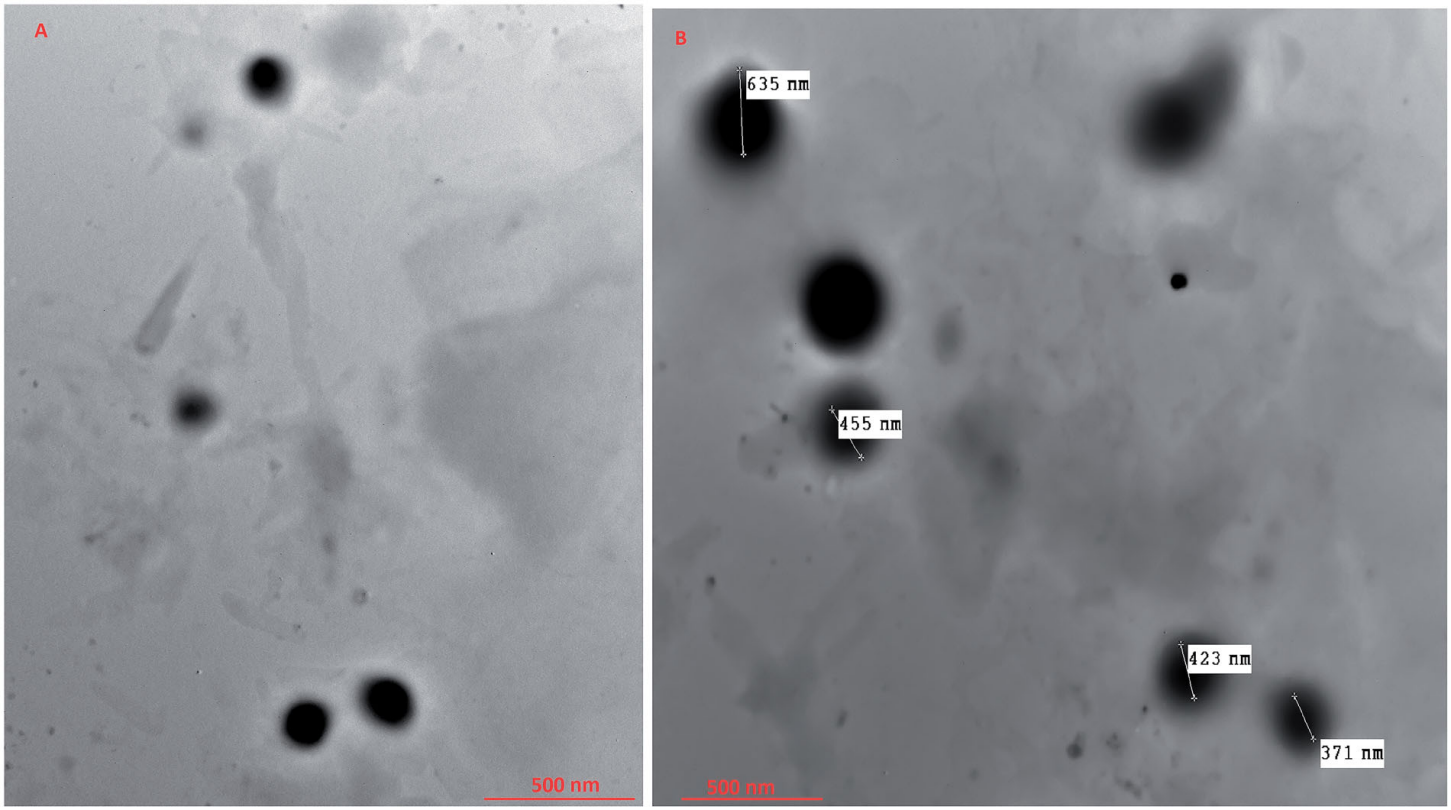
TEM micrographs of the optimized VCZ loaded MLNCs formulation.

TEM micrographs of the optimized VCZ loaded MLNCs exhibited spherically shaped, non-aggregating nanoparticles, Figure 4. The optimized VCZ loaded MLNCs showed comparable nano-size to that formerly gained from the Malvern particle size analyzer, Table 2.

#### 3.4.3. Ph, viscosity and spreadability measurements

The pH of the optimized VCZ loaded MLNCs was found to be 4.55 ± 0.26 which is considered slightly acidic. This pH is acceptable in the case of vaginal preparations in which the physiological pH of the vagina is between 3.8 and 4.5, and hence the irritation potential due to the difference in pH is supposed to be minimal.

**Figure 5.**
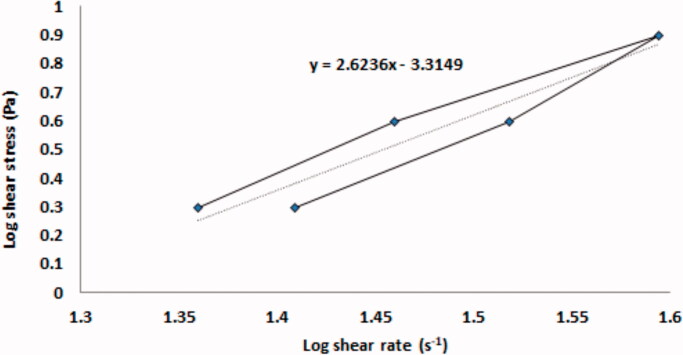
The rheogram of the optimized VCZ loaded MLNCs formulation, showing the thixotropic behavior of the prepared formulation.

The viscosity of the optimized VCZ loaded MLNCs was found to be 964.49 ± 57.99 cp. The rheogram of the optimized VCZ loaded MLNCs is shown in Figure 5. The calculated furrow’s constant (N) for the optimized formulation was found to be 2.6236 indicating a shear-thinning system. This is a desirable pattern in the case of semisolid dosage forms, as it indicates the breakdown of the 3D network structure upon applying shear stress. This thixotropic behavior would facilitate the preparation process, in addition, the pouring, the spreading, and the handling would meet the patient acceptance. The spreadability test indicates the easiness of application of the semisolid formulation, and hence the success of the formulation adhesion to the vaginal mucosa. The spreadability was calculated and was found to be 24.55 ± 1.32 g.cm.sec^−1^, demonstrating good spreading characteristics of the optimized formulation and suggesting the formation of a coherent film covering the vaginal mucosa, which prolongs the contact time and improves treatment outcomes.

#### 3.4.4. Bioadhesion study

Bioadhesion potentiality of the optimized VCZ loaded MLNCs was determined by the vertical displacement test, where the distance moved by the formulation from the top to the bottom of the agar/mucin gel plate during the test period was measured. The optimized formulation was not displaced under the force of gravity. The results confirmed the potentiality of the formulation to stick to the vaginal wall, and hence increase the contact time to allow the delivery of the drug to the infected tissues and thus improve the efficacy of the treatment.

#### 3.4.5. *In vitro* drug release and stability studies

**Figure 6.**
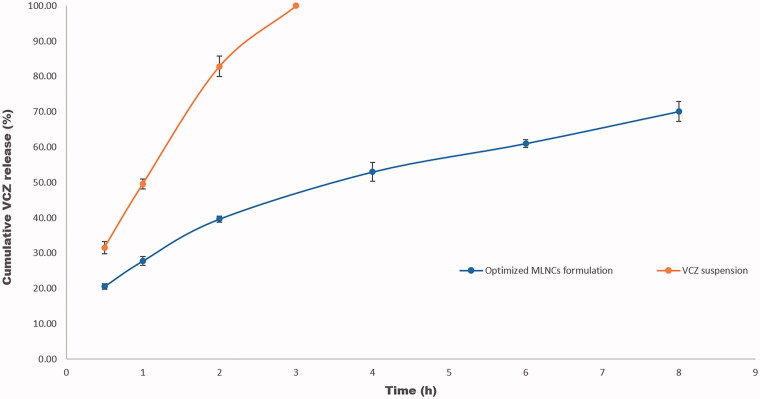
Release profiles of VCZ from the optimized MLNCs formulation and VCZ aqueous suspension in acetate buffer (pH 4, simulating vaginal pH) containing 0.5% Tween^®^ 80 at 37 ± 0.5 °C.

VCZ release profile from the optimized MLNCs formulation displayed sustained drug release behavior, where 70.08% was released after 8 hr, Figure 6. On fitting the release data to different kinetic models, *R^2^* values obtained were 0.8812, 0.9667, 0.9943, and 0.8034 for zero-order, first-order, Higuchi model, and Korsmeyer Peppas, respectively. From the data, it was deduced that the release behavior of the optimized VCZ loaded MLNCs followed the Higuchi model. Similar results were obtained by Pradhan et al. upon formulating nanostructured lipid carriers containing fluocinolone acetonide for topical treatment of psoriasis (Pradhan et al., 2015).

The stability study of any developed formulations is one of the most crucial principles and requirements for any dosage form. Upon storage at different temperatures, the optimized VCZ loaded MLNCs showed a non-significant change in EE %, PS, and PDI (*p* > .05). Furthermore, no substantial change (*p* > .05) was observed in viscosity, Q_8_, pH, and spreadability, confirming the stability of the optimized formulation, Table 3.

**Table 3.** Characteristics of the optimized VCZ loaded MLNCs before and after storage at (6 ± 2 °C) and (25 ± 2 °C) for 3 months.

Optimized MLNCs	EE (%)	PS (nm)	PDI	Viscosity (cp)	Q_8_ (%)	pH	Spreadability
Before storage	67.16 ± 3.45	322.72 ± 15.11	0.22 ± 0.03	964.49 ± 57.99	70.08 ± 2.87	4.55 ± 0.26	24.55 ± 1.32
After storage at (6 ± 2 °C) for 3 months	65.87 ± 1.33	317.43 ± 10.66	0.21 ± 0.01	951.40 ± 69.32	72.39 ± 3.76	4.50 ± 0.39	23.36 ± 0.83
After storage at (25 ± 2 °C) for 3 months	64.67 ± 3.86	329.78 ± 8.54	0.24 ± 0.02	973.00 ± 76.65	69.84 ± 2.47	4.58 ± 0.11	24.22 ± 0.59

MLNCs: mixed-lipid nanoconstructs; EE%: Entrapment efficiency percentage; PS: particle size; PDI: polydispersity index; Q8: the cumulative percent of VCZ released after eight hours.

All values are represented as mean ± standard deviation (*n* = 3).

### 3.5. *In vivo* investigations

#### 3.5.1. Evaluation of the antifungal activity

**Figure 7.**
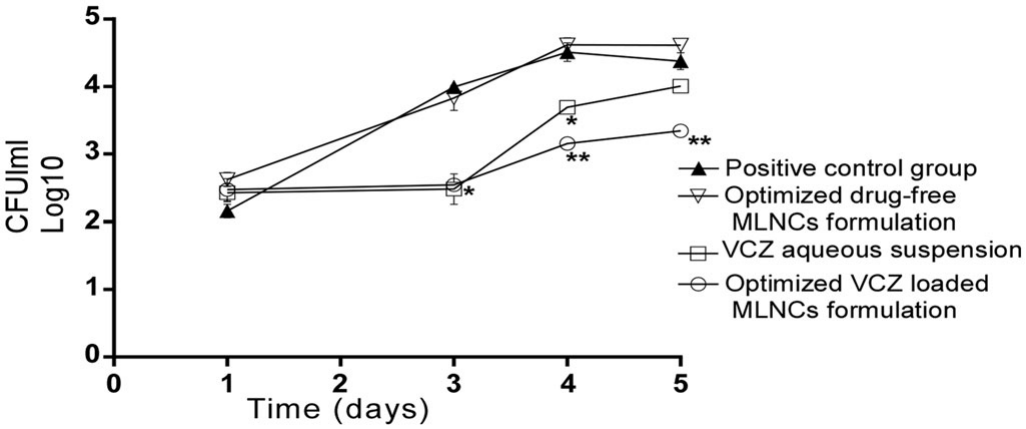
Microbiological observations of therapeutic efficacy of the optimized VCZ loaded MLNCs formulation, drug-free MLNCs formulation, and VCZ suspension against vaginal candidiasis in female Wistar rats. Data represented as the mean of log_10_ CFU/ml and the error bars represent the standard error (*n* = 5). The first day on the graph corresponds to the third day of inoculation. Statistical analysis was performed by applying analysis of variance (ANOVA) at *p*-value < .05. The * marks the significant repression in log_10_ CFU/ml compared to the control. The ** marks the significant repression in log_10_ CFU/ml compared to the drug suspension.

Before the beginning of the treatment, swabs were taken from the rats’ vagina and then were cultured to confirm the infection with *C. Albicans* through counting the number of CFU. The outcomes illustrated in Figure 7 showed the existence of yeast cells with no significant difference (*p* < .05) among all groups on the third day of induction of vaginal candidiasis and before receiving any treatment (represented as the first day on the graph). Twenty-four hours following the treatment application (represented by the third day on the graph), there was no significant difference in CFU/mL between the groups treated with the optimized VCZ loaded MLNCs formulation and VCZ aqueous suspension. However, forty-eight and seventy-two hours following the treatment application (represented by the fourth and fifth day on the graph, respectively), VCZ loaded MLNCs formulation significantly reduced the fungal burden compared to the aqueous VCZ suspension, the optimized drug-free MLNCs formulation as well as the control group (*p* < .05). This could be explained in light of the enhanced entrapment of VCZ inside MLNCs that sustained the release of the drug, besides the proper consistency of the formulation that allowed the retention of the drug in the vaginal cavity.

#### 3.5.2. Histopathological study

**Figure 8.**
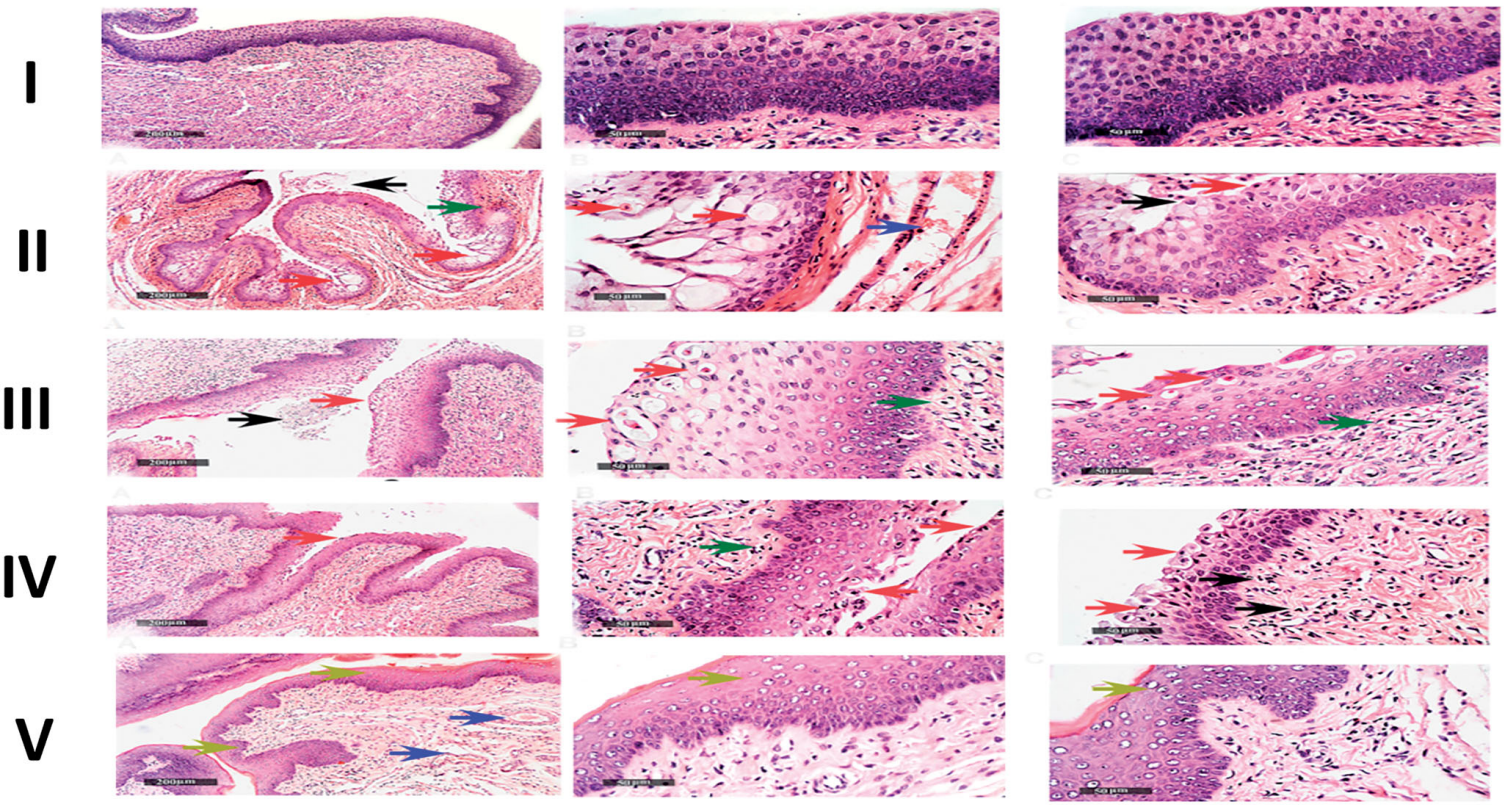
Microscopical histopathological examinations of rats with vaginal candidiasis 7 days after infection stained by Hematoxylin and Eosin. I. Section of negative control rat vagina showing normal morphological features of the vaginal mucosa with almost intact lining epithelium. II. Section of positive control rat vagina showing apoptotic vaginal keratinocytes (red arrow), intraluminal desquamated epithelial cells (black arrow), congestion and dilatation of submucosal blood vessels (blue arrow) as well as subepithelial inflammatory cells infiltrate (green arrow). III. Section of rat vagina treated with the optimized drug-free MLNCs showing the same records as the positive control samples. IV. Section of rat vagina treated with VCZ suspension showing degenerative changes in superficial layers of vaginal keratinocytes (red arrow) with occasional intraluminal desquamated cells (black arrow), subepithelial inflammatory cells infiltrates (green arrow), and minimal records of congested submucosal blood vessels. V. Section of rat vagina treated with the optimized VCZ loaded MLNCs showing normal morphological features of the vaginal mucosa with almost intact lining epithelium (yellow arrow) and nominal congested submucosal blood vessels (blue arrow).

The histopathological findings were found to coincide with the fungal burden results. The microscopical examination of the vaginal rat samples of the negative control group (I) displayed normal morphological features of the vaginal mucosa with almost

intact lining epithelium, as well as submucosal layers with minimal inflammatory cells, infiltrate records, and normal vasculatures, Figure 8(I). The positive control group (group II) showed wide areas of degenerative changes in the epithelium lining of the vaginal mucosa, in addition to intraluminal desquamated epithelial cells. Moderate congestion and dilatation of submucosal blood vessels were also observed as well as subepithelial inflammatory cells infiltrate, Figure 8(II). The group treated with drug-free MLNCs formulation (group III) showed the same records as the positive control group, Figure 8(III). Regarding the group treated with the aqueous suspension of VCZ (group IV), most of the samples showed persistence records of degenerative changes in the superficial layers of the vaginal keratinocytes with occasional intraluminal desquamated cells. Minimal records of congested submucosal blood vessels were reported. However, moderate infiltration with subepithelial inflammatory cells was observed, Figure 8(IV). On the other hand, the histopathological changes in the group treated with the optimized VCZ loaded MLNCs formulation (group V) showed well-protected normal morphological features of the vaginal mucosa, where minimal inflammatory cells infiltrate and nominal congested submucosal blood vessels were observed, Figure 8(V). For better data visualization and interpretation, comparative scored histopathological findings are listed in Table 4.

**Table 4.** Comparative histopathological study of the rat groups treated with drug-free MLNCs formulation (group III), VCZ aqueous suspension (group IV), and VCZ loaded MLNCs (group V), versus the uninfected negative control (group I) and the untreated positive control (group II).

Histopathological findings	Negative control (Group I)	Positive control (Group II)	Drug-free MLNCs formulation (Group III)	VCZ aqueous suspension (Group IV)	VCZ loaded MLNCs formulation (Group V)
Degenerative changes	–	+++	+++	++	_
Inflammatory cell infiltrates	–	+++	+++	++	+
Congested blood vessels	–	++	+	_	–

+++: Severe; ++: Moderate; +: mild; –: nill.

## 4. Conclusion

MLNCs were successfully formulated using low lipids concentrations, consisting of mixtures of solid lipid and oils, applying high shear homogenization and ultrasonication. MLNCs were stabilized by Tefose 63 and Gelot 64. A full 2^4^ factorial design was exploited for the optimization of MLNCs. The optimized formulation displayed nano-spherical particles, with high VCZ EE%, and showed thixotropic consistency with high bioadhesivity. The *in vivo* studies revealed that the incorporation of VCZ into MLNCs reduced the fungal burden, in female Wistar rats infected with vaginal candidiasis, compared to the aqueous VCZ suspension (*p* < .05). Furthermore, the histopathological findings proved the effectiveness of the optimized formulation in the management of VVC. Consequently, the obtained results suggest that the formulated dosage form can be considered as a potential nano-scaled lipid-based carrier for improving the delivery of VCZ through the vaginal mucosa. Further clinical studies are planned to authenticate the effectiveness of the optimized formulation in the management of VVC.
